# Autoimmunity and Infection in Glomerular Disease

**DOI:** 10.3390/microorganisms11092227

**Published:** 2023-09-02

**Authors:** Chiara Casuscelli, Elisa Longhitano, Veronica Maressa, Silvia Di Carlo, Luigi Peritore, Simone Di Lorenzo, Vincenzo Calabrese, Valeria Cernaro, Domenico Santoro

**Affiliations:** Unit of Nephrology and Dialysis, Department of Clinical and Experimental Medicine, A.O.U. “G. Martino”, University of Messina, 98122 Messina, Italy; elisa.longhitano@libero.it (E.L.); maressaveronica15@gmail.com (V.M.); silviadicarlo1@yahoo.it (S.D.C.); luigiperitore1994@gmail.com (L.P.); simodilorenzo92@gmail.com (S.D.L.); v.calabrese@outlook.it (V.C.); valeria.cernaro@unime.it (V.C.)

**Keywords:** infection, glomerulonephritis, autoimmunity, bacteria, virus, parasites

## Abstract

The ongoing glomerular damage of infections is not limited to the most widely known form of post-streptococcal glomerulonephritis, which is today less common in the Western world; other forms of glomerulonephritis are associated with several bacterial, viral and parasitic pathogens. The mechanisms responsible range from the direct damage of glomerular cells to the formation and deposition of immunocomplexes to molecular mimicry to the secretion of superantigens. Similarly, in the course of glomerular disease, infections are more frequent than in the general population due to the loss of immunoglobulins in urine and the immunosuppressive agents used to treat the autoimmune disease that decrease the activity of the immune system. Recognizing this two-way link, understanding its pathogenetic mechanism, and identifying the most appropriate therapeutic choice are essential for the personalized management of patients. In this continuously developing field, this short review summarizes the current state of the art as support for physicians, who are increasingly involved in managing patients with glomerular disease and infections.

## 1. Introduction

Autoimmunity represents an abnormality of the immune system in which self-components of the body become target antigens. The mechanisms that generate it are complex and largely still unknown. Genetic susceptibility, environmental factors, and infections appear to be involved.

In the kidney, in addition to the direct cytotoxic effects of pathogens, host humoral and cellular defense mechanisms activated by an infectious process can cause glomerular damage by forming in situ immunocomplexes or deposition of circulating immunocomplexes [1].

A recognized mechanism is molecular mimicry, whereby a similarity between exogenous antigens and self-antigens triggers an autoimmune response against self-structures with a similar amino acid sequence [1,2].

The production of superantigens (viral or bacterial peptides that directly activate T cells by binding to their domain Vβ) can also play a role in determining a massive cell-mediated response with subsequent production of polyclonal IgG that can generate autoimmune responses [1]. Superantigens, in addition, can be assisted by some molecules released locally due to the infectious inflammatory process (such as uric acid or fragments of bacterial or viral DNA) that stimulates the activation of immune cells [1].

Moreover, infection-dependent local damage can result in conformational changes in proteins with exposure to components recognized as non-self that activate the autoimmune response by acting as epitopes [1].

The link between autoimmunity and infection in kidney damage is enriched yearly with new evidence, and this short review aims to synthesize the characteristics of various microorganisms implicated in glomerular kidney damage, highlighting the gaps in etiopathogenesis that still need to be filled.

## 2. Bacterial Infections and Glomerulonephritis

Glomerulonephritis from bacterial infection (Table 1) may be a clinical sequel to a previous bacterial infection or manifest during an acute or chronic infectious process. Several causative agents may be responsible.

Streptococcus pyogenes, a group A Streptococcus (GAS), is the most significant pathogen. The incidence of GAS infections is reduced in developed countries due to improved hygiene and health surveillance systems [3]. Acute post-streptococcal glomerulonephritis (APSGN) is one of the complications of this bacterial infection. Typically, it appears 1–2 weeks following pharyngitis and 4–6 weeks following impetigo. APSGN occurs mainly in childhood, with a peak of incidence between 3 and 12 years of age, with a male predominance; in adulthood, it is more frequent in the elderly population [4]. The clinical presentation is variable, ranging from an asymptomatic form associated with microhematuria to rapidly progressive glomerulonephritis. When symptomatic, findings include nephritic syndrome, characterized by micro/macrohematuria, proteinuria that can also reach the nephrotic range, edema, arterial hypertension, and acute renal failure. In 90% of cases, usually at an initial phase of the disease, C3 and CH50 reduction are observed, returning to normal within 4–8 weeks.

The host predisposition and microorganism virulence factors favor the onset of the disease. In particular, the genetic polymorphisms HLA-DP and HLA-DR-B are associated with APSGN onset [5,6,7]. Streptococcal antigens involved in immunopathogenesis lead to the formation of circulating or in situ immune complexes. Furthermore, specific nephritogenic streptococcal antigens can activate the alternate complement pathway and inflammatory response by an increase in chemotactic factors and IL-6 in the mesangium [8]. Protein M is a bacterial surface protein capable of stimulating antibody production after cross-reaction with glomerular antigens. It is encoded by the EMM gene, and the molecular typing of GAS strains is based on its genetic differences. The strains more related to the APSGN onset are 1, 2, 12, 49, 55, and 73 EMM GAS types [9]. Streptococcal pyrogenic exotoxin B (SpeB) is a cationic cysteine proteinase that is found in the subepithelial deposits; it determines the destruction of the basal membrane by the complement, pro-collagenases and metalloproteinases activation [10]. Nephritis-associated plasmin receptor (NAPIr) is a glycolytic enzyme with a glyceraldehyde-3-phosphate dehydrogenase (GAPDH) activity; it has a plasmin-like activity that may promote a local inflammatory reaction [11]. Both NAPIr and SPEB have been shown in renal biopsy after SGA infection, and high antibody titers are found in more than 90% of recovering patients [12]. Other antigens implicated are streptokinase, streptococcal histone-like proteins, and streptococcal enolase [13].

Streptococcus mutans is a facultative anaerobic bacterium, a significant pathogen of human dental caries. A few studies presented an association with the onset of IgA Nephropathy. In particular, S. mutans strains with the collagen-binding protein (Cnm) are related to increased adhesion and invasion of the epithelium and the development of systemic diseases [14]. However, other studies are needed to make the pathogenesis clearer.

Staphylococcus aureus is a commensal bacterium colonizing approximately 30% of the human population. It is the etiological agent of many clinical manifestations, from relatively benign skin and soft tissue infections (impetigo, folliculitis, cellulitis) to life-threatening conditions, such as endocarditis, osteomyelitis, meningitis, and toxic shock syndrome [15]. It also contributes to the development of some autoimmune illnesses.

Staphylococcal-infection-associated glomerulonephritis (SAGN) is constantly increasing in developed countries. Hemminger et al. and Nasr collected the most numerous cases of SAGN; in both, there was a marked predominance in the males, with the average age of onset being 55–58 years [16,17]. The disease was linked to intravenous drug abuse in younger populations exposed to infectious risk. Other risk factors included diabetes, liver cirrhosis, alcoholism, and malignancy. Kidney damage onsets when the triggering infection is still in the phase of activity. It may occur in most cases with nephritic syndrome associated with acute kidney injury, microhematuria (rarely macrohematuria), and/or proteinuria, sometimes in the nephrotic range. Laboratory tests frequently show hypocomplementemia (C3 with or without reduction of C4), which tends to normalize after about two months from onset, except in cases of persistent infection [18].

Staphylococcal antigens, such as enterotoxins, may act as superantigens, activating T cells, which in turn activate B cells that produce polyclonal IgA, IgG, and IgM [19].

S. aureus infection may also be responsible for GN related to infective endocarditis in intravenous drug users. Patients have low serum complement C3 or C4, ANCA and ANA positivity, and cryoglobulinemia that can cause pulmonary hemorrhage mimicking anti-GBM disease [20].

Shunt nephritis, caused by Staphylococcus aureus, Staphylococcus epidermidis, and Staphylococcus albus, is a complication of chronic infection on ventriculoatrial, ventriculi jugular, or ventriculoperitoneal shunts inserted for the treatment of hydrocephalus that cause a rare immune complex glomerulonephritis [20].

The IgA-dominant infection-related GN (IgA-DIRGN) is an immune-complex-mediated disease described as concomitant with methicillin-resistant Staphylococcus aureus (MRSA), methicillin-sensitive Staphylococcus aureus and other infections such as E. coli, Streptococcus epidermidis, and Klebsiella [20]. In this case, staphylococcal enterotoxin B serves as a superantigen that induces immunological hyperactivation with T cell activation and related stimulation of B cells and polyclonal IgA production. The predominance of IgA glomerular deposition with dominant or codominant C3 is the characteristic finding on the immunofluorescence of renal biopsy.

Some studies have shown the relationship between the nasal carrier status of Staphylococcus aureus and the development of granulomatosis with polyangiitis (GPA), a subtype of ANCA-associated vasculitis. The pathogenesis is unclear: S. aureus may express a PR-3-like peptide, causing GPA with a molecular mimicry, or it may release neutrophil extracellular traps (NETs) that transfer antigens to dendritic cells [21,22].

Treponema pallidum is a bacterium from the order Spirochaetales; it is the causative microorganism of syphilis, a sexually and vertically transmitted disease with an increased incidence in recent years. Renal damage is rare. Membranous nephropathy is the more frequent renal pathology, presenting with nephrotic syndrome and rarely associated with other systemic signs such as rash or lymphadenopathy [23]. Other glomerular lesions have been reported, including minimal change disease, focal segmental glomerulosclerosis, membranoproliferative glomerulonephritis, and crescentic glomerulonephritis [24]. The histological examination shows the presence of treponemal antigens and antibodies which indicate an immune complex damage [25].

Helicobacter pylori (HP) is a Gram-negative and urease-positive bacterium, which colonizes gastric mucosa determining gastritis, ulcer disease, and gastric cancer [26]. At the renal level, it can cause IgA vasculitis (IgAVN) [27]. The role of HP in the pathogenesis of IgA vasculitis is unclear: it seems to be involved in the abnormal glycosylation of IgA1 and the deposition of immune complexes [28]. A study demonstrated the capacity of cytotoxin-associated gene A (CagA) to stimulate abnormal glycosylation of IgA1; another study demonstrated high IgA titer in the serum of patients with an active HP infection [29,30]. However, the role of H. pylori remains uncertain.

Tropheryma whipplei is a Gram-positive bacillus and a causative antigen of Whipple’s disease (WD). Direct renal involvement has not been well recorded in the literature [31]. Some authors hypothesize that kidney damage occurs late in the course of WD or, probably, the urogenital system is more resistant [32]. Nonspecific segmental glomerulonephritis and IgA nephropathy have been described [33,34]. Recently, a case of phospholipase A2-receptor-positive membranous glomerulonephritis has been reported [35]. In this case, the subepithelial deposits and the organisms highlighted with phospholipase A2 receptor immunohistochemical stain, suggesting that the circulating PLA2R is hijacked by T. whipplei and, after phagocytosis by macrophages, leads to a development of an autoimmune response [35].

Bartonella species are Gram-negative bacteria that cause a wide range of clinical manifestations, such as cat scratch disease, bacillary angiomatosis, and infective endocarditis. In about 40–50% of cases of infective endocarditis with renal damage, kidney failure may occur. The spectrum of kidney lesions is large; clinical cases of crescentic glomerulonephritis and pauci-immune glomerulonephritis have been reported [36,37]. Various clinical cases reported the relation between ANCA vasculitis and Bartonella endocarditis; the pathogenetic mechanism of the ANCA formation is unclear [38].

### Renal Morphological Changes Linked to Bacterial Infections

Whatever the causative bacterial agent, the most common morphological characteristic found under the light microscope in glomerular damage related to bacterial infection is endocapillary hypercellularity with a prominent neutrophil infiltrate that configures acute exudative glomerulonephritis. Glomerular involvement can be diffuse and global or focal and segmental; in some cases, it is possible to highlight the crescents (especially in post-endocardial glomerulopathy) and a slight mesangial hypercellularity typical in the resolution of the disease [39]. At the intracapillary level, we can find hyaline pseudo thrombi.

Glomerular injury is often associated with tubulointerstitial damage with the development of acute interstitial nephritis or acute tubular necrosis, both characterized by a neutrophil infiltrate that may mimic acute pyelonephritis [20].

Immunofluorescence shows C3 infiltration in the capillary walls and the mesangium with or without accompanying immunoglobulins. The staining shows a granular pattern which may have a “garland” (deposits along the capillary walls, typical of the active phase of the disease), “starry sky” (widespread deposits on the capillary wall and in the mesangium), or “mesangial” (mainly mesangial deposits) distribution [20].

The accompanying immunoglobulins can be IgG in the post-streptococcal form, predominantly IgA and K chains in the staphylococcal forms, or predominantly IgM in the shunt forms or associated with endocarditis [20].

Electron microscopy shows the characteristic sign represented by electron-dense subepithelial deposits called “humps”. The “humps” prefer the mesangial folds or mesangial waist zones. They have a broad base and project into the overlying podocytes (which are activated and enlarged). They are more present in the active phase of the disease and in staphylococcal forms and less common in the resolution phase or the chronic forms and in shunt and post-endocardial forms [40].

## 3. Viral Infections and Glomerulonephritis

Various viral infections may be a cause of glomerulonephritis (Table 2).

EBV, also known as human herpesvirus 4 (HHV4), is a ubiquitous virus that latently infects much of the world’s population. Infectious mononucleosis is the most frequent clinical manifestation, but renal damage is not rare [41].

An association with systemic lupus erythematosus, favored by mechanisms of molecular mimicry (antibodies against the EBNA-1 protein that react in a cross-reaction with the dsDNA), appears to emerge from the studies [42]. Anti-EBNA-1 antibodies can also deposit in the glomerules and induce proteinuric damage, as shown in experiments on mice [43]. The role of direct harm has yet to be made clear. EBV infection has also been associated with IgA nephropathy (IgAN) in genetically predisposed individuals [44].

Hepatitis B virus (HBV) is a DNA virus of the hepadnavirus family. HBV is responsible for acute and chronic liver infections, which in their evolution lead to hepatocellular carcinoma. HBV is known to be an infectious trigger for secondary membranous nephropathy (MN), and it has been found that HbeAg forms IgG-HBeAg immune complexes, which are deposited in the glomerular subepithelial space [45,46]. A study evaluating the difference between HBV-MN and HBV-complicated primary MN showed that immunoglobulins deposited in the kidney were different, with IgG1 deposition in HBV-MN and IgG4 deposition in primary MN [47]. Furthermore, vaccination programs have been shown to reduce the incidence of HBV-MN [48] and antiviral treatment reduces proteinuria and induces complete remission in many patients with HBV-MN [49]. It is important to differentiate primary NM from HBV-MN because immunosuppressive treatment exacerbates the infection in HBV-infected patients. Other morphological rarer patterns such as membranoproliferative glomerulonephritis (MPGN), minimal change disease (MCD), and IgA nephropathy have been described [50].

Hepatitis C virus (HCV) is an RNA virus that can lead to liver fibrosis and hepatocellular carcinoma. Chronic HCV infection also induces extrahepatic manifestations, among which are cryoglobulinemia and cryoglobulinemic vasculitis (CV) [51]. In 30% of cases of cryoglobulinemia, renal involvement is characterized by MPGN, which represents the main renal HCV-related alteration [52]. Clinically, renal involvement manifests as nephrotic or non-nephrotic proteinuria, microscopic hematuria, and refractory hypertension.

HCV infection is hypothesized to trigger an upregulation of B-cell-activating factor (BAFF), inducing clonal expansion of B lymphocytes and the formation of aberrant antibodies [53]. HCV also activates complement with decreased C4 levels. Since effective antiviral therapy against HCV was introduced, the prevalence of HCV-CV has dramatically decreased. However, some patients maintain cryoglobulin levels despite viral eradication. It is thought that HCV-mediated B-cell proliferation could become autonomic, with cryoglobulin production and disease induction. This seems to be supported by the persistence of t(14;18) positive B cell clones and small amounts of HCV-RNA in the lymphatic system, even after antiviral therapy [54,55]. Although not as strongly correlated with HBV infection, HCV has also been considered a possible trigger for the development of membranous nephropathy (MN) [56]. The pathogenetic mechanism is not clear, HCV could infect the kidney itself, or there may be an immune response against viral antigens and immune complex deposition, as with HBV [56,57]. Human immunodeficiency virus (HIV) is an RNA virus belonging to the Retroviridae family that infects and reduces the CD4+ T helper. Today, infection control rarely leads to acquired immune deficiency syndrome (AIDS). Kidney disease remains a common consequence of HIV infection [58]. HIV-associated kidney disease can be divided into HIV-associated nephropathy (HIVAN) and HIV-immune complex renal disease (HIVICK) [45]. HIVAN results from a direct viral infection of renal epithelial cells and podocytes, resulting in collapsing glomerulopathy with active tubulointerstitial inflammation [59,60]. The most typical presentation is acute renal failure with proteinuria [61]. Among the predisposing risk factors are the APOL1 factor, especially in the African American population, males, and those with various comorbidities such as diabetes mellitus and hypertension. HIVK results from the deposition of immune complexes in the glomerulus and from tissue damage, but the mechanism by which this occurs is not well known: circulating immune complexes can remain trapped in the glomerulus, or immune complexes can be formed in situ. Several HIVICK-associated glomerulopathies have been described in the literature, including lupus-like glomerulonephritis, HIV-associated IgA nephropathy, and HIV-associated membranous nephropathy [62,63,64,65]. ANCA antibodies (AAV-associated) and anti-GBM antibodies (anti-GBM disease-associated) have been found in HIV-infected patients without any renal disease, raising the suspicion that the formation of autoantibodies in HIV may not always be pathogenic [66,67,68,69]. In the HIVAN, antiviral treatment reports to the normal podocyte phenotype, while in the HIVICK, the specific therapy is ineffective in treating the disease or slowing its progression, but further study is needed to determine the pathogenesis and efficacy of treatment.

Parvovirus B19 (PVB19) is a DNA virus belonging to the Parvoviridae family. It can generally cause arthropathy, infectious erythema and transient aplastic crises in predisposed individuals such as in subjects with sickle cell anemia. It can also determine the formation of autoantibodies and cause autoimmune diseases. There are SLE-like features in acute PVB19 infection (fever, myalgias, rash, lymphadenopathy, hypocomplementemia, and the presence of antinuclear antibodies), so a patient’s condition is thought to be associated [70]. The exact pathogenesis is unknown but NS1, a PVB19 helicase, may play a role: NS1 has been shown to bind to dsDNA and a study by Puttaraksa et al. demonstrated that NS1 induces dsDNA autoantibody formation and SLE-like disease in an animal model [71,72]. In this context, PVB19 infection would cause a lupus-like syndrome by inducing the formation of immune complexes [73]. Parvovirus infection is also linked to the development of renal vasculitis, particularly Henoch Schonlein purpura, anti-GBM disease, and granulomatosis with polyangiitis [74,75]. However, very few case reports were identified, so a precise link could not be established.

Severe acute respiratory syndrome coronavirus 2 (SARS-CoV-2) is an RNA virus belonging to the Coronaviridae family that emerged in 2019 and has caused the COVID-19 pandemic that has spread worldwide, causing the death of more than 5.7 million people from 2019 to February 2022. Regarding renal manifestations, recent studies highlight a 12.3% incidence of acute kidney injury (AKI) in patients with COVID-19 [76]. Proteinuria and hematuria are frequent findings. Histologically, tubular lesions and necrosis are the most common manifestations, followed by the collapsing variant of glomerulopathy and extensive podocyte lesions. Furthermore, some studies have shown that copies of SARS-CoV-2 have been identified in the kidney tissue and in the urine of infected patients, suggesting that direct infection may participate in kidney damage [77,78]. SARS-CoV-2 infection has also been associated with the development and recurrence of other known autoimmune renal syndromes. Cases of IgA nephropathy, IgA vasculitis, ANCA vasculitis and anti-GBM disease have been reported [79,80,81,82,83,84,85,86,87,88,89]. However, the pathogenetic mechanism or any pre-infection genetic predisposition is not known. Therefore, further clinical studies are needed to elucidate the pathophysiology behind the association between SARS-CoV-2 and autoimmunity, as many questions are still open.

## 4. Parasitic Infections and Glomerulonephritis

Parasitic infections are known to be a frequent cause of disease in humans, affecting millions of people each year, particularly in tropical and subtropical areas characterized by low levels of hygiene and sanitation (Africa, Asia, and Latin America). Over time, parasitic infections have increasingly emerged as an important cause of kidney disease and can cause acute kidney injury, glomerulonephritis, and tubular dysfunction. Parasitic infections that cause glomerulonephritis include malaria, schistosomiasis, and filariasis (Table 3) [90].

Malaria is the leading cause of morbidity and mortality in tropical and subtropical areas where this disease is endemic (sub-Saharan Africa, Southeast Asia, and South America). Malaria is caused by four species of the genus Plasmodium: Plasmodium vivax, Plasmodium falciparum, Plasmodium malaria, and Plasmodium ovale. Renal involvement generally presents as a form of AKI, especially in cases of P. falciparum infection (62.5%), which can cause acute tubular necrosis (NTA) [91]. Other renal manifestations include acute infection-related glomerulonephritis, acute interstitial nephritis, and chronic and progressive glomerulonephritis. The latter, at the renal biopsy, can reveal five stages: mild focal and segmental, moderate focal and segmental, diffuse or segmental lesions with interstitial and tubular changes, marked sclerosis, and interstitial/tubular atrophy. This lesion is very infrequent and, in the past, has been know as “quartan malarial nephropathy” (QMN) [20].

Schistosomiasis is a significant parasitic disease of humans in terms of morbidity and mortality. Schistosoma mansoni, Schistosoma haematobium, and Schistosoma japonicum are the major schistosomes infecting humans. Humans contract schistosomiasis when they come into contact with water sources contaminated with the infectious form of the parasite (cercaria). The eggs hatch, releasing miracidia that penetrate the snails (intermediate host), wherein they undergo asexual reproduction. The cercarial forms are released, penetrate the skin and reach the liver via the bloodstream, where they mature to adulthood and reside in the mesenteric or perivesical venous plexus [92]. The parasite eggs release proteolytic enzymes and cause inflammation and ulceration of the bladder and ureters. Dysuria, hematuria, pollakiuria, and proteinuria are typical manifestations of the disease. Chronic infection can lead to fibrosis of the bladder and ureters; this represents a significant risk factor in the development of carcinoma. Renal involvement is observed in a limited number of cases (about 5–6%). Schistosomal glomerular disease derives from an immune response by the host against the schistosome eggs. The clinical glomerular disease has been described most frequently in association with hepatosplenic schistosomiasis produced by S. mansoni. This generally appears asymptomatic, presenting only more or less severe proteinuria, hematuria, and rarely hypocomplementemic acute nephritis. Renal histology shows a form of mesangioproliferative or membranoproliferative glomerulonephritis and less frequent membranous nephropathy, focal segmental glomerulosclerosis, and amyloidosis. The AFRAN association of nephrology described five patterns of schistosomal glomerular pathology, and a sixth pattern has been added to describe the form associated with HCV coinfection: mesangioproliferative, proliferative exudative, membranoproliferative, focal segmental glomerulosclerosis, amyloidosis, cryoglobulinemia. The symptomatic form is related to hepatic fibrosis from S. mansoni, an independent risk factor for developing chronic glomerulopathy due to liver macrophage dysfunction and decreased immune complex clearance [20].

Filariasis is a disease caused by an infection by nematodes of the Filariidae family, commonly known as “filariae”, endemic in many parts of south-east Asia, including India.

Filarial worms are nematodes transmitted to humans through a mosquito vector and dwell in the subcutaneous tissues and lymphatics. The parasite has been isolated from many body sites, commonly subcutaneous tissue, epididymis, and spermatic cord. Rarely, it has been demonstrated from lymph nodes, urine, thyroid, pericardium, and pleura.

Renal disease is infrequent, and glomerular involvement is also unlikely. When presenting renal glomerular damage, light microscopy reveals diffuse proliferative MPGN, MCD, chronically sclerosing GN, or the collapsing variant of FSGS. It is possible to directly detect microfilariae in the arterioles, glomerular and peritubular capillary lumina, tubules, and interstitium and/or immune deposits along with worm antigens and structural components with immunofluorescence and electron microscopy. Clinically, the kidney disease manifests with urinary abnormalities: proteinuria and/or hematuria were detected in cases with lymphatic filariasis. Nephrotic syndrome occurs especially with polyarthritis and chorioretinitis [20].

## 5. Treatment of Infection

Based on the concept that understanding the pathogenetic mechanisms causing glomerular damage should guide treatment and the studies showing that infection-related glomerulonephritis responds well to infection control itself, with antibiotics, or with antivirals, the KDIGO 2021 guidelines suggest the use of targeted antibiotic therapy in bacterial forms with positive cultures and recommend the use of nucleoside analogs in HBV-related glomerulonephritis (HBV DNA > 2000 IU/mL) and antiretroviral treatment in all patients with HIV and kidney disease [20,93,94].

The role of antibiotic therapy is different in the various infections due to glomerular diseases. In SAGN, antibiotic treatment must be timely to eradicate the infection [3]. In APSGN, the antibiotics are not indicated during nephritis but at the time of GAS infection to prevent nephritis onset [3]. The prophylactic role of antibiotic therapy is especially important in countries with a high incidence of GAS infection [3]. Also, extending prophylaxis to the contacts of affected patients makes it possible to further reduce transmission and epidemics [3].

The benefit of corticosteroids or other immunosuppressive drugs in infection-related glomerulonephritis remains uncertain. The KDIGO 2021 guidelines recommend avoiding drugs such as cyclophosphamide and rituximab in cases associated with active HBV infection because they can promote virus replication [20].

Studies are also underway to assess the efficacy of target therapy for the interferon-APOL1 cycle in carriers of high-risk APOL1 alleles [94,95].

**Figure 1:**
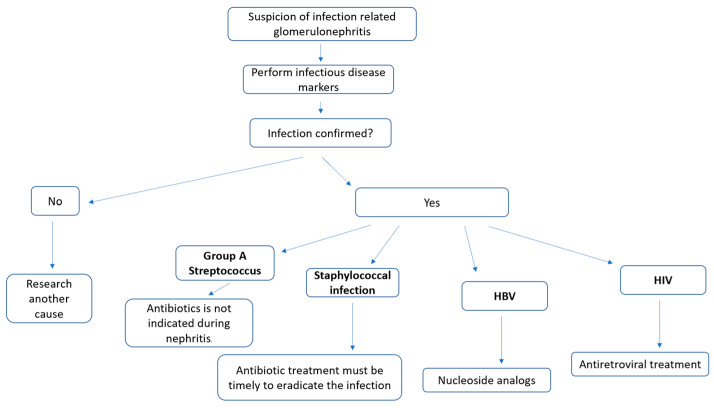
Algorithm of management in suspicion of infection-related glomerulonephritis.

The management in suspicion of infection-related glomerulonephritis is schematized in the Figure 1.

## 6. Immunosuppressive Treatment and Infection

Beyond being the cause of glomerulonephritis, infections can sometimes represent one of the most fearsome complications. The increased risk of infection, especially in glomerulonephritis presenting as nephrotic syndrome, has several reasons. First of all, the fluid collection represents an optimal growth ground, while loss of immunoglobulins in urine can impair the ability to eliminate microorganisms. Finally, immunosuppressive agents commonly and successfully used to treat such autoimmune diseases act by decreasing the activity of the immune system.

Corticosteroids have been broadly used in the treatment of glomerulonephritis. They act through inhibition of T-cell activation by blocking transcription of NFkB and through induction of lymphopenia by stimulating the movement of lymphocytes into lymphoid tissue. Infectious complications include bacterial infection, viral infection, such as varicella-zoster virus and herpes virus, and reactivation of latent tuberculosis or hepatitis [96]. However, the risk of infection is not increased if the daily prednisone dose is less than 10 mg or if the cumulative dose does not exceed 700 mg [97]. At the same time, patients receiving once-daily administration should be at lower risk than those receiving multiple administrations [98]. Conversely, the risk of infection is highly increased if hypoalbuminemia is present, a common finding in patients with nephrotic syndrome. To prevent such complications, close follow-up during therapy is needed, in addition to vaccination against pneumococcus and influenza and careful examination of skin and mucosa to detect mycosis or candidiasis early [96].

Clinical trials that foresaw the use of high-dose corticosteroids have not infrequently been interrupted for the high incidence of infectious complications [99].

Cyclophosphamide is an alkylating agent with antiproliferative action on T and B cell proliferation. It is used mainly in systemic lupus erythematosus, ANCA-associated vasculitis, and proliferative glomerulonephritis. The most frequent infections related to its use are community-acquired pneumonia, herpes zoster, soft tissue infection, catheter-related infection, and pyelonephritis, besides cytomegalovirus. Increased susceptibility to infection was also correlated in a retrospective study by Cavallasca et al. with a time of use and cumulative doses; particularly, a cut-off of 20g of the cumulative dose was established, and beyond this value, there was a six times greater chance of infection [100]. CMV infection is a common complication of patients treated with cyclophosphamide, and lower eGFR is a risk factor for developing such infection; moreover, CMV-infected patients have a higher need for renal replacement therapy. Unlike in transplant recipients, currently, there is no consensus about treating CMV in GN patients; this infection is correlated to the cessation of immunosuppressive therapy and, consequently, to the worsening of glomerular disease. Hence, prophylactic therapy might be considered. A single-center prospective study in Turkey proposed a cut-off of 500 copies of CMV DNA per ml to treat CMV infection and improve outcomes in glomerulonephritis patients [101]. Furthermore, a risk stratification algorithm was proposed by Lim et al. to decide who deserved antiviral agents among patients with GN with positive CMV [102]. Oral vanganciclovir or intravenous ganciclovir was administered if at least three of the following criteria were present: eGFR < 15 mL/min, use of methylprednisolone, cyclophosphamide, mycophenolate, Rituximab or plasma exchange. In treated patients, there were fewer cases of infection, although more therapy-related adverse events were reported.

Rituximab (RTX) is an anti-CD20 monoclonal antibody that depletes B cells. Initially used in treating non-Hodgkin’s lymphoma, it has subsequently progressed as a treatment of numerous glomerular diseases such as membranous nephropathy, minimal change disease, focal segmental glomerulosclerosis, and lupus nephritis. Trivin et Al. found an incidence of severe infection of 21.6 per 100 patient-years in patients with glomerulonephritis receiving RTX [103]. The frequency of infection worsened as kidney function declined and with the use of other immunosuppressive drugs; conversely, a relatively safe infection profile was shown in patients treated with RTX only. More importantly, the severity of infection and death correlated with a cumulative dose of Rituximab, while other studies proved that a single administration of RTX is relatively safe [104]. Most reported infections were bacterial, particularly pneumonia, thus highlighting the importance of Streptococcus pneumonia and Haemophilus influenza vaccination before RTX administration. Moreover, in this study, various patients presented with rare opportunistic infections like candidiasis, aspergillosis, or CMV, thus suggesting that antibody-mediated response might play a role against such organisms that were originally thought to be controlled by innate or T cell immune response. Moreover, patients treated with RTX have an increased risk of hepatitis reactivation [105].

Mycophenolate mofetil and its activated form mycophenolic acid inhibit monophosphate dehydrogenase, which plays a fundamental role in synthesizing guanosine nucleosides, a pivotal pathway for T and B lymphocyte proliferation. It is a standard of care in lupus nephritis due to its clinical efficacy and safety profile; among the side effects is an increased risk of infection. The most reported sites of infection include the respiratory tract (upper respiratory tract, bronchus), urinary tract, skin, and eye [106].

Tacrolimus, a calcineurin inhibitor, showed superiority compared to ciclosporin in terms of efficacy and safety and has found a place in the treatment of primary membranous nephropathy, lupus nephritis, and FSGS. Compared to cyclophosphamide, tacrolimus showed no significant difference in infection rates [107]. Compared to other drugs used to treat lupus nephritis, fewer infections were reported; in juvenile patients, there were some reported cases of herpes virus infection bronchitis [108].

Eculizumab, a humanized monoclonal antibody, plays its role in binding the C5 complement component, thus blocking complement activation. Among kidney diseases, it finds its place in the treatment of atypical hemolytic uremic syndrome. Unluckily, this helpful drug can increase the risk of infection from microorganisms such as Neisseria gonorrhoeae and Neisseria meningitidis [109]. Consequently, patients treated with Eculizumab, or other C5 inhibitors, should receive antimicrobial prophylaxis for the whole course of therapy plus meningococcal vaccination [109].

## 7. Conclusions

Infection can be the cause or the trigger of glomerular disease, and, in the same way, glomerulonephritis can favor microbial colonization. Although research always offers us new knowledge in this field, further studies are needed to better understand the link between infection and glomerulonephritis, allowing us to provide the best personalized care to patients. The current challenge should be based on the primary prevention of infections through the extension of vaccination campaigns, hygiene measures, and the containment of high-risk behaviors for contagion; adequate prevention would limit the use of antibiotics, and therefore, the correlated nephrotoxicity and antibiotic resistance. Moreover, techniques for the rapid localization of the infection, like the nuclease-activated probe for detecting S. Aureus infection described in the literature in studies on animals, could be developed. Another field in which research should be conducted is drugs that reduce bacterial biofilm formation.

## Figures and Tables

**Table 1:** Bacterial infections and glomerulonephritis.

Species	Pathogenesis	Glomerular Disease	Clinical Manifestation
Streptococcus species	In situ immune complexes formation or circulating immune complexes deposition	Acute post-streptococcal glomerulonephritis (APSGN)	Nephritic syndrome
Staphylococcus aureus	Bacterial superantigens; Immune complexes deposition (IgA and C3 dominant); Molecular mimicry	Staphylococcal infection-associated glomerulonephritis (SAGN); ANCA associated vasculitis	Nephritic syndrome
Treponema pallidum	In situ immune complexes formation or circulating immune complexes deposition	Membranous nephropathy	Nephrotic syndrome
Helicobacter pylori	Abnormal glycosylation of IgA1 and mesangial deposition	IgA vasculitis	Nephritic syndrome
Tropheryma whipplei	Circulating immune complexes deposition	Membranous nephropathy	Nephrotic syndrome
Bartonella species	Unclear	ANCA associated vasculitis	Nephritic syndrome

**Table 2:** Viral infections and glomerulonephritis.

Species	Pathogenesis	Glomerular Disease	Clinical Manifestation
Epstein–Barr virus	Molecular mimicry; Abnormal glycosylation of IgA1 and mesangial deposition.	Lupus nephritis; IgA nephropathy	Nephritic syndrome
Hepatitis B virus	In situ immune complexes formation or circulating immune complexes deposition	Membranous nephropathy MPGN MCD IgA nephropathy	Nephrotic syndrome Nephritic or nephrotic syndrome
Hepatitis C Virus	Circulating immune complexes deposition	Cryoglobulinemic vasculitis MPNG	Nephritic or nephrotic syndrome
Human immunodeficiency virus	Direct renal cell infection; In situ immune complexes formation or circulating immune complexes deposition.	HIV-associated nephropathy (HIVAN); HIV-immune complex renal disease (HIVICK).	Acute Renal Failure; Nephritic or nephrotic syndrome
Parvovirus B19	Circulating immune complexes deposition	Lupus-like syndrome Henoch Schonlein purpura Wegner’s granulomatosis	Proteinuria
Severe acute respiratory syndrome coronavirus 2 (SARS-CoV2)	Direct renal cell infection; Immune-mediated renal damage	Collapsing glomerulopathy and acute tubular necrosis; IgA nephropathy, IgA vasculitis, ANCA vasculitis, anti-GBM disease	Acute renal failure; Nephritic syndrome

**Table 3:** Parasitological infections and glomerulonephritis.

Species	Pathogenesis	Renal Damage	Clinical Manifestation
Plasmodium	Immune complexes formation and complement activation; Direct renal cell infection;	Acute infection-related glomerulonephritis; Acute tubular necrosis or acute interstitial nephritis; Quartan malarial nephropathy (focal and segmental or diffuse lesions)	Nephritic syndrome; Acute renal failure; Nephrotic syndrome.
Schistosoma	Immune-mediated damage	Mesangioproliferative or membranoproliferative nephropathy	Nephritic or nephrotic syndrome
Filariae	Direct renal cell infection	Mesangioproliferative glomerulonephritis, minimal change disease or focal segmental glomerulosclerosis	Nephritic or nephrotic syndrome
